# Development of an automated method to detect sitting pivot transfer phases using biomechanical variables: toward a standardized method

**DOI:** 10.1186/1743-0003-9-7

**Published:** 2012-02-03

**Authors:** Guillaume Desroches, Martin Vermette, Philippe Gourdou, Dany Gagnon

**Affiliations:** 1 School of Rehabilitation, University of Montreal, Montreal, Canada; 2 Pathokinesiology Laboratory, Center for Interdisciplinary Research in Rehabilitation of Greater Montreal, Institut de réadaptation Gingras-Lindsay-de-Montréal, Canada

**Keywords:** Activities of daily living, kinetics, kinematics, rehabilitation, spinal cord injury, task performance and analysis, upper extremity

## Abstract

**Background:**

Sitting pivot transfer (SPT) is one of the most important, but at the same time strenuous at the upper extremity, functional task for spinal cord injured individuals. In order to better teach this task to those individuals and to improve performance, a better biomechanical understanding during the different SPT phases is a prerequisite. However, no consensus has yet been reached on how to depict the different phases of the SPT. The definition of the phases of the SPT, along with the events characterizing these phases, will facilitate the interpretation of biomechanical outcome measures related to the performance of SPTs as well as strengthen the evidence generated across studies.

**Methods:**

Thirty-five individuals with a spinal cord injury performed two SPTs between seats of similar height using their usual SPT technique. Kinematics and kinetics were recorded using an instrumented transfer assessment system. Based on kinetic and kinematic measurements, a relative threshold-based algorithm was developed to identify four distinct phases: pre-lift, upper arm loading, lift-pivot and post-lift phases. To determine the stability of the algorithm between the two SPTs, Student *t*-tests for dependent samples were performed on the absolute duration of each phase.

**Results:**

The mean total duration of the SPT was 2.00 ± 0.49 s. The mean duration of the pre-lift, upper arm loading, lift-pivot and post-lift phases were 0.74 ± 0.29 s, 0.28 ± 0.13 s, 0.72 ± 0.24 s, 0.27 ± 0.14 s whereas their relative contributions represented approximately 35%, 15%, 35% and 15% of the overall SPT cycle, respectively. No significant differences were found between the trials (p = 0.480-0.891).

**Conclusion:**

The relative threshold-based algorithm used to automatically detect the four distinct phases of the SPT, is rapid, accurate and repeatable. A quantitative and thorough description of the precise phases of the SPT is prerequisite to better interpret biomechanical findings and measure task performance. The algorithm could also become clinically useful to refine the assessment and training of SPTs.

## 1. Background

The large number of sitting pivot transfers (SPTs) performed daily by individuals with a spinal cord injury, along with the excessive physical strains acting on the wrist, elbow and shoulder joints while carrying out this functional task likely contributes to the development or perpetuation of secondary upper extremity musculoskeletal impairments over time in this population [1]. Such a potentially damaging cycle deserves attention given the relevance of preserving U/E integrity and optimizing performance in this population. Comprehensive biomechanical assessments of SPTs have been shown to be a useful approach to gain greater insight into the performance of this task and to increase scientific evidence needed for clinical practice changes. However, the fact that no consensus has yet been reached on how to depict the different phases of the SPT task limits the strength of the evidence. Allison [2] and his collaborators [3-5] first defined the pre-transfer adjustment, dynamic and re-balancing phases using kinetic data. Perry et al, [6] referred to the preparation, lift and descent phases using a combination of elbow motions and visually determined event markers to identify when the buttocks lost contact with the initial seat and landed on the target seat. Nawoczenski et al., [7] proposed the preparatory, lift/pivot and sit-back phases using a combination of angular and linear movements of the thorax segment. Gagnon et al., [8] described the pre-lift, lift and post-lift phases using a combination of kinematic and kinetic measures [9-12]. Although all these methods have led to valuable outcome measures for SPTs, comparing the results across these various studies remains difficult.

As previously reported [2], efforts should initially be made to refine the definition of the SPT phases and the events characterizing these phases in order to facilitate the interpretation of biomechanical outcome measures related to the performance of SPTs and to strengthen the evidence generated across studies. Using a standardize definition of SPT phases would enable researchers to compare results between studies and highlight deficits during specific phases which could in turn be clinically useful to refine the assessment and training of SPTs. Therefore, the main objective of this study was to develop and test a relative threshold-based algorithm to automatically detect four distinct SPT phases using kinematic and kinetic event markers.

## 2. Methods

### 2.1. Participants

A convenience sample of thirty-five individuals (gender: 32 males/3 females; age: 43.5 ± 10.9 years of age; height: 1.80 ± 0.10 m; weight: 78.8 ± 17.3 kg) who sustained a complete or incomplete sensorimotor SCI at various vertebral levels (third thoracic vertebra to fourth lumbar vertebra) on average 11.1 ± 10.6 years prior to the study were recruited. The participants used a manual wheelchair as their primary means of locomotion (≥ 4 hours/day) and were able to perform SPTs between two surfaces independently without the use of a technical aid. Ethical approval was obtained from the Research Ethics Committee of the Centre for Interdisciplinary Research in Rehabilitation of Greater Montreal (CRIR-541-0810). Participants reviewed and signed an informed consent form prior to entering the study.

### 2.2. Kinetics

**Figure 1:**
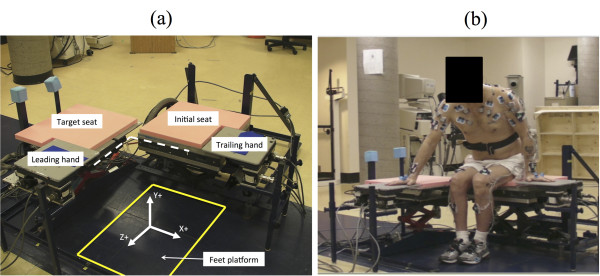
**Instrumented assessment system used**. (a) Five force-sensing devices are placed strategically under both seat, hands and feet. (b) An example of a subject equipped with the LEDS.

An instrumented transfer assessment system that incorporates five separate force-sensing surfaces to measure the reaction force underneath the feet, buttocks (initial and target seats) and hands (leading and trailing) during the SPTs was used (Figure 1a). Two height-adjustable instrumented chairs were positioned beside one another with a 90° angle separating the two seats and were set at a height similar to the participant's wheelchair (mean height = 0.42 ± 0.02 m). The hand force-sensing surfaces, attached laterally to each chair, were adjusted to replicate the width of the participant's wheelchair seat. All forces applied on these surfaces were continuously recorded, amplified and stored at a sampling frequency of 600 Hz during the SPTs. Subsequently, the forces recorded during these tasks were filtered using a fourth-order Butterworth zero-lag filter, with a cut-off frequency of 10 Hz, and were down-sampled to 60 Hz. This instrumented transfer assessment system has been described in a previous report [13].

### 2.3. Kinematics

Kinematic parameters during the SPTs were recorded at a sampling frequency of 60 Hz, using a motion capture system consisting of six synchronized camera units (4 Optotrak model 3020 and 2 Optotrak Certus camera units; NDI Technology Inc., Waterloo, Ontario, Canada). This system tracked the 3D trajectory of 60 non-collinear skin-fixed light emitting diodes (LEDs) placed on the rigid bodies, defining the head, trunk, upper and lower limbs. In addition, specific bony landmarks were digitized using a 6-marker probe to further define articular centers and principal axes of segments [11]. The marker coordinates were smoothed with a fourth-order Butterworth zero-lag filter using a cut-off frequency of 6 Hz. Custom-made programs were used to quantify kinematic parameters (joint angle, velocity and acceleration) at the shoulder and elbow joints [11].

### 2.4. Sitting pivot transfers

From a quiet seated position (starting position) with the hands placed on the force-sensing surfaces (starting position), participants were instructed to perform SPTs between the initial and target seats (Figure 1a). Participants used their natural technique to achieve the SPTs, especially in terms of movement amplitude and velocity. Participants performed two SPTs with the right upper limb (dominant upper limb for all participants), which acted as the leading upper limb during the SPTs as it was positioned on the instrumented target hand surface.

### 2.5. Definition of SPT event markers and phases

**Figure 2:**
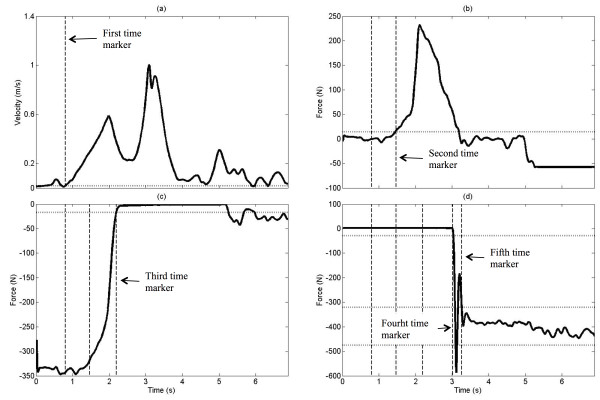
**Time series components of the kinematic and kinetic data used in the threshold-based algorithm**. (a) Evolution of the linear velocity of the C7 LED; the dotted line represent the threshold (2 SD of the quiet sitting) and the dashed line represent the first time marker that satisfied the condition. (b) Evolution of the vertical force under the trailing hand; the dotted line represent the 5% threshold and the dashed line the second time marker. (c) Time series of the vertical force underneath the initial seat; the dotted line the 5% threshold and the dashed line the third time marker. (d) Evolution of the vertical force under the target seat; the upper dotted line represent the 5% threshold whereas the two lower dotted line represent the mean ± 2 SD of the vertical force during the post-lift phase. Both fourth and fifth time markers are displayed (dashed lines).

To define the different phases of the SPT and the relative threshold-based algorithm, a combination of kinetic and kinematic data was used, namely the norm of the time derivative of the 3D trajectory of the spinous process of the 7^th ^vertebra (C7), the vertical components of the reaction force recorded underneath the trailing hand force-sensing surface and the initial and the target seats. The four phases proposed in the present method are based on three phases previously defined by Gagnon et al., [9] in which the pre-lift phase has been refined to include an upper limb-loading phase. The beginning of the pre-lift phase, which also defines the start of the SPT, is determined when the linear velocity of the C7 process LED exceeds 2 standard deviations of the starting position for more than half a second (Figure 2a). The standard deviation is computed from the first 20 frames of the trial during which the subject is seated quietly. The subsequent and thus new phase of the SPT, i.e., the upper-limb loading phase, is determined by the vertical force under the trailing hand. The beginning of the upper-limb loading phase is defined by the first frame of the rapidly rising vertical force applied under the trailing hand, which exceeds 5% of the maximum force recorded underneath this hand during the complete transfer cycle for more than half a second (Figure 2b). The start of the lift-pivot phase is when the vertical force under the initial seat falls below 5% of the maximum force applied for more than one second (Figure 2c). The post-lift phase begins when the vertical force under the target seat exceeds 5% of the maximum force recorded for more than one second (Figure 2d). Finally, the end of the SPT is determined by the vertical force under the target seat, which must remain within 2 standard deviations of the mean vertical force recorded during the post-lift phase (Figure 2d). All event markers used to automatically define the start and finish of the four different phases of the SPTs were identified using the relative threshold-based algorithm programmed into a custom-built Matlab routine (See additional file).

### 2.6. Statistics

For each participant, the absolute and relative duration of each of the four phases along with the total absolute duration were determined for each of the two SPTs performed using the relative threshold-based algorithm, and averaged to obtain mean values (± 1 SD). Then, group mean values (± 1 SD) were computed for all these variables. To determine the stability of the algorithm between the two SPTs performed by each participant (within-subject difference), Student *t*-tests for dependent samples were performed on the absolute time of each phase for both trials using SPSS^® ^for Windows (version 11.5) and a level of significance of 0.05 was selected.

## 3. Results

**Figure 3:**
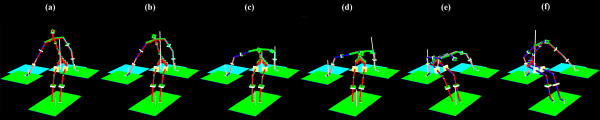
**Schematic reconstruction of one subject**. (a) at rest before SPT, (b) at the start of the pre-lift phase, (c) at the start of the upper-limb loading phase, (d) at the start of the lift-pivot phase, (e) at the start of the post-lift phase and (f) at the end of the SPT cycle.

The mean (± 1 SD) absolute time for all phases of each of the two SPT trials as well as the mean for both SPT trials combined are summarized in Table 1. The relative phase duration (i.e., percentages) of each phase are also presented in Table 1. Similar phase duration (p > 0.05) across the two SPT trials was confirmed. The two longest phases are the pre-lift and lift-pivot phases (0.74 s and 0.72 s) and, on average, correspond to 37% and 36% of the entire SPT cycle, respectively. The upper limb-loading phase, i.e., the new phase introduced in this paper, lasts on average 0.28 ± 0.13 s and typically represents 14% of the entire SPT. Figure 3 shows the position of one subject for each time event.

**Table 1:** Mean (1 SD) duration of each SPT phase for trial 1, trial 2 and the average of both trials along with the relative percentage of each phase

Phases	Time (s)			*P*-values	%
		
	Trial 1	Trial 2	Average		
Pre-lift	0.72	0.75	0.74	0.652	37.0
	(0.35)	(0.39)	(0.29)		

Upper limb loading	0.29	0.27	0.28	0.480	14.0
	(0.16)	(0.15)	(0.13)		

Lift-pivot	0.72	0.72	0.72	0.891	36.0
	(0.28)	(0.24)	(0.24)		

Post-lift	0.26	0.27	0.27	0.707	13.0
	(0.13)	(0.17)	(0.14)		

SPT cycle	1.99	2.01	2.00	0.843	100
	(0.59)	(0.61)	(0.49)		

## 4. Discussion

The approach proposed in this study for analyzing SPTs provides researchers with a means of standardizing the reporting of biomechanical research findings comparable to the methods that currently exist for gait [14,15], manual wheelchair propulsion [16,17] and sit-to-stand studies [18,19]. Considering the widely varying approaches used to define the phases in previous studies, adhering to a common framework seems essential, especially due to the growing interest in biomechanical studies relating to SPT tasks. Consequently, new knowledge will most likely emerge because comparison across studies will become possible and collaborations among research teams will be facilitated as a result of this common definition for SPT. Adhering to a common framework has been highly valuable in expanding the corpus of knowledge on gait and wheelchair biomechanics for examples. Moreover, the proposed method also enables researchers to time-normalize the SPT cycle to 100 data points (100%) with varying relative contribution of the pre-lift (35%), upper arm loading (15%), lift-pivot (35%) and post-lift phases (15%) to the overall SPT cycle.

This study confirms that the use of a relative threshold-based algorithm to automatically detect four distinct phases of the SPT, aside from the quiet siting position that precede the SPT, based on kinematic and kinetic event markers is rapid, accurate and repeatable, while analyzing results of biomechanical assessment of SPTs. Using quantitative measurements to identify phases and events during SPTs will enable researchers and clinicians to identify troublesome events during SPTs. Moreover, the implementation of the proposed relative threshold-based algorithm allows researchers to refine the description of four distinct phases during SPTs, including a newly defined upper limb-loading phase. The latter strengthens the biomechanical analysis of SPTs as it represents a rapid transition (mean duration = 0.28 s) during which the upper limbs, especially the trailing one, are exposed to rapidly increasing and substantial loads since the buttocks raise off the initial seat and start to move towards the target one. It was essential to isolate this phase during SPTs as it has been documented that rapidly rising force (i.e., the rate of rise of force) may precipitate secondary musculoskeletal impairments to upper limb joints [20,21]. The lift phase, during which the buttocks are not supporting any weight, is easily identifiable and also highly demanding for both upper limbs and lasts over twice as long than the upper limb-loading phase (mean duration = 0.72 s). Thus, these two phases definitively deserve special attention as they yield useful insight into a possible secondary musculoskeletal impairment mechanism during SPTs in individuals with SCI.

A potential limitation is the fact that sophisticated laboratory equipment was used in the present study to define the kinematic and kinetic parameters of the SPT, essential to automatically identify the event markers that define the phases of the SPT. Allison et al., [4,5] used a single force platform and video recording to study movement strategies of individuals with SCI. Perry et al., [6] used only video recordings in order to study the electromyographic activity of the upper arm muscles during SPTs. More recently, Koontz et al., [22] used two force platforms and retro reflective markers to evaluate upper limb joints kinetics during SPTs. Thus, various method and equipment are used to study SPTs. The implementation of the relative threshold-based algorithm could be easily achieved with any of the aforementioned methods. Alternatively, adding a miniature accelerometer positioned at C7 and pressure sensors placed underneath hands and buttocks, may be suitable to use with the relative threshold-based algorithm to depict the phases of the SPT.

## 5. Conclusion

The relative threshold-based algorithm used automatically detected the four distinct phases of the SPT that were defined. The algorithm was shown to be quick, reliable and repeatable. A quantitative and thorough description of the precise phases of the SPT is prerequisite to better interpret biomechanical findings and measure task performance. The algorithm could also become clinically useful to refine the assessment and training of SPTs.

## Competing interests

The authors declare that they have no competing interests.

## Authors' contributions

GD participated in the design and coordination of the study, took part in the data acquisition, analysis and interpretation, conducted the statistical analyses and drafted the manuscript. MV participated in the coordination of the study and data acquisition and revised the manuscript. PG participated in data acquisition and analysis and revised the manuscript. DG participated in the concept and design of the study, data acquisition, analysis and interpretation, helped in the draft and revision of the manuscript, obtained funding and supervised the study. All the authors red and approved the final version of the manuscript.

## Supplementary Material

Additional file 1 **Pseudo-code to determine sitting pivot transfer phases**. This file contains a pseudo-code algorithm to determine each of the five time markers for the sitting pivot transfer cycle. Click here for file
