# Supplementary material for: Development of an automated method to detect sitting pivot transfer phases using biomechanical variables: toward a standardized method
Source: J Neuroeng Rehabil. 2012 Feb 3;9:7. doi: 10.1186/1743-0003-9-7 (PMC3298704; doi:10.1186/1743-0003-9-7)
Supplement: Additional file 1 — Pseudo-code to determine sitting pivot transfer phases. This file contains a pseudo-code algorithm to determine each of the five time markers for the sitting pivot transfer cycle. [file 1743-0003-9-7-S1.PDF]

```
function intervals = findPhasesTransfers(O,FTH,FIS,FTS,freq)
% Description: Pseudo code of threshold-based algorithm under Matlab script
%
% Inputs:      - O(n x 1 vector)   = vector sum of the linear velocity of C7 process
%                                    trajectory
%              - FTH(n x 1 vector) = vertical force measured under the trailing hand
%              - FIS(n x 1 vector) = vertical force measured under the initial seat
%              - FTS(n x 1 vector) = vertical force measured under the target seat
%              - freq(scalar)      = acquisition frequency (60 Hz in the current
%                                    experiment)
%
% Outputs:     - intervals (m x 1) = vector containing the frame number for the
%                                    beginning of each phase

% First time marker (beginning of the pre-lift phase)

n = size(O,1); % number of frames for the velocity vector
MO = mean(O(1:20,1)); % mean for the first 20 frames of the velocity vector
StdO = std(O(1:20,1)); % standard deviation for the first 20 frames of the velocity
                       % vector

% Loop to identify the first frame that is outside 2SD
for i = 1:n
    if(O(i,1) > MO+2*StdO || O(i,1) < MO-2*StdO)
        % When the first frame is indentified, the subsquent frame responding to the
        % same criteria are found until more than half second (freq/2) as passed
        cmp = 1;
        while((O(i+cmp,1) > MO+2*StdO || O(i+cmp,1) < MO-2*StdO) && cmp <= freq/2)
            cmp = cmp + 1;
        end
        % If the time criterion is met, than the beginning frame is found and the
        % algorithm exit the for loop
        if(cmp > freq/2)
            break;
        else
            i = i + cmp;
        end
    end
end
% The frame of the beginning of the pre-lift phase is attributed into the output vector
intervals(1,1) = i;

% Second time marker (beginning of the upper-arm loading phase)

FTHn = abs(FTH)./max(abs(FTH)); % the vertical force under the trailing hand is
                                % normalize to its max

% We search for the frames that are under 5% of the maximal value, by doing so, it's
% going to easier to identify the gap between the frames that are excluded (ones that
% responds to the actual criteria for that phase) to see if the met the time criteria
% (half a second)
ii = find(FTHn < 0.05);
i = find(diff(ii) >= freq/2);
% The frame for the beginning of the upper-arm loading phase is attributed to the
% output vector
intervals(2,1) = ii(i(end));

% Third time marker (beginning of the sitting-pivot phase)

FISn = (abs(FIS))./max(abs(FIS)); % the vertical force under the initial seat is
                                  % normalize to its max

% We search for the frames that are under 5% of the maximal value, by doing so, it's
% going to easier to identify the gap between the frames that are excluded (ones that
% responds to the actual criteria for that phase) to see if the met the time criteria
```

```
% (a second)
ii = find(FISn < 0.05);
i = find(diff(ii) >= freq);

% The frame for the beginning of the sitting pivot phase is attributed to the output
% vector
intervals(3,1) = ii(i(end));

% Fourth time marker (beginning of the post-lift phase)
FTSn = (abs(FTS))./max(abs(FTS));

% We search for the frames that are under 5% of the maximal value, by doing so, it's
% going to easier to identify the gap between the frames that are excluded (ones that
% responds to the actual criteria for that phase) to see if the met the time criteria
% (a second)
ii = find(FTSn < 0.05);
i = find(diff(ii) >= freq);
% The frame for the beginning of the post-lift phase is attributed to the output vector
intervals(4,1) = ii(i(end));

% Fifth time marker (end of the sitting pivot transfer cycle)

% Only the vertical force from the beginning of the post-lift phase to the end of the
% trial is taken
FTSt = FTS(intervals(4,1):end,1);
MF = mean(FTSt); % mean of the vertical force
StdF = std(FTSt); % standard deviation of the vertical force

% Loop to identify the frame that meet the criteria
for i = 1:size(FTSt,1)
    if(FPSt(i,1) <= MF+2*StdF && FPSt(i,1) >= MF-2*StdF)
        % When the first frame is identified, the subsequent frame responding to the
        % same criteria are found until more than a second (freq) as passed
        cmp = 1;
        while(FPSt(i+cmp,1) <= MF+2*StdF && FPSt(i+cmp,1) >= MF-2*StdF && cmp <= freq)
            cmp = cmp + 1;
        end
        % If the time criteria is met, than the beginning frame is found and the
        % algorithm exit the for loop
        if(cmp > freq )
            break;
        else
            i = i + cmp;
        end
    end
end
% The frame for the beginning of the post-lift phase is attributed to the output vector
intervals(5,1) = i;
```
